# Oral Findings in Hemodialyzed Patients Diagnosed with Diabetes Mellitus and/or Hypertension—A Systematic Review

**DOI:** 10.3390/jcm12227072

**Published:** 2023-11-13

**Authors:** Agata Trzcionka, Dagmara Mączkowiak, Rafał Korkosz, Mansur Rahnama, Jan Duława, Marta Tanasiewicz

**Affiliations:** 1 Department of Conservative Dentistry with Endodontics, Faculty of Medical Sciences in Zabrze, Medical University of Silesia, Plac Akademicki 17, 41-902 Bytom, Poland; 2 Department of Dental Surgery, Medical University of Lublin, Karmelicka 7, 20-081 Lublin, Poland; 3 Department of Internal Medicine and Metabolic Diseases, School of Health Sciences in Katowice, Medical University of Silesia, 40-752 Katowice, Poland

**Keywords:** hemodialysis, hemodialyzed patients, end-stage chronic kidney disease, oral status, periodontal status, oral hygiene, mucosa, saliva

## Abstract

Chronic kidney disease is classified as a civilization disease and is being diagnosed in an increasing number of patients. Hypertension and diabetes mellitus often coexist in hemodialyzed patients. The aim of the present study was to identify publications on the oral cavity status of multimorbid hemodialyzed adult patients additionally diagnosed with hypertension and/or diabetes mellitus, published between 2012 and 2022 to establish evidence of the impact of hypertension and diabetes mellitus on the oral status of hemodialyzed patients. Scopus and Web of Science databases were searched. Eight articles were included in the review. In total, 3 articles discussed oral hygiene in hemodialyzed patients, 4 discussed periodontal status, 3 discussed mucosa condition and saliva parameters, and 3 discussed the problem of Candidiasis infections. The conclusions were as follows: there is still a limited number of publications discussing the oral status of hemodialyzed patients diagnosed with hypertension; involved articles have proven that coexisting diseases can influence the oral cavity status of hemodialyzed patients and cause periodontal disorders, lower hygiene status, saliva parameters and make the risk of Candida infections higher.

## 1. Introduction

Chronic kidney disease (CKD) is caused by the gradual reduction in nephron functionality that leads to the loss of the kidneys’ function [1]. Chronic disease is characterized by progression, high morbidity, and high death rates of affected patients. It very often is diagnosed in people suffering from diabetes mellitus and hypertension [2]. In end-stage chronic kidney disease, when the glomerular filtration rate (gFR) is lower than 15 mL/min/1.72 m^2^, patients are at risk of potentially lethal complications, which is why, in that group of patients, kidney replacement therapy (KRT) (hemodialysis, peritoneal dialysis or transplantation) is recommended [3]. Almost 4 million people are undergoing kidney replacement therapy, the most popular of which is hemodialysis (69% of KRT), and 89% are undergoing all dialysis [4].

There are some publications available describing the poor condition of the oral cavity in hemodialyzed patients [5,6,7,8]. Researchers observed poor oral hygiene (increased accumulation of dental plaque, and presence of dental calculus), pathological changes in periodontal tissues (gingiva overgrowth, higher incidents of gingivitis, and periodontitis), changes in saliva parameters (decreased salivary secretion, increase in its density), taste disorders and halitosis [8].

Many researchers have also investigated the correlation between diabetes mellitus and oral cavity status. The most frequently observed oral complications were an increased frequency of caries occurrence, xerostomia, periodontal diseases in the form of gingivitis and periodontal disease, taste disorders, and burning mouth syndrome. Diabetic patients were also more prone to infections [9].

Medications used in the treatment of hypertension may lead to gingival overgrowth, xerostomia, salivary gland swelling, lichenoid reactions, taste disorders, and parathesis [10]. Most side effects are observed in therapy with the following group of medications: angiotensin-converting enzyme inhibitors, calcium channel blockers, and diuretics [11].

Chronic kidney disease is classified as a civilization disease. It affects more and more people. In 2017, 850 million people were diagnosed with chronic kidney disease. Hemodialysis is the most common out of the available kidney replacement therapy methods (69% of all kidney replacement therapies and 89% out of all dialysis). Results of the cross-sectional study in 2018 showed that the median country-specific use of hemodialysis was 298.4 per million in the population [4]. There is still insufficient dental care provided for hemodialyzed patients, and they lack information on how important dental care is in the treatment of chronic kidney disease and coexisting pathologies.

The aim of our study was to identify publications regarding the oral status of hemodialyzed patients suffering from diabetes mellitus and/or hypertension published between 2012 and 2022 (with the usage of Web of Science and Scopus databases) and establish the evidence of the impact of hypertension and diabetes mellitus on the oral status of hemodialyzed patients. We aimed to assess the comparability of the chosen studies. The authors also wanted to assess how the experience of particular members of our research team influenced the conducting of particular stages of the review process.

## 2. Materials and Methods

### 2.1. Strategy of Searching and Criteria of Material Selection

**Figure 1:**
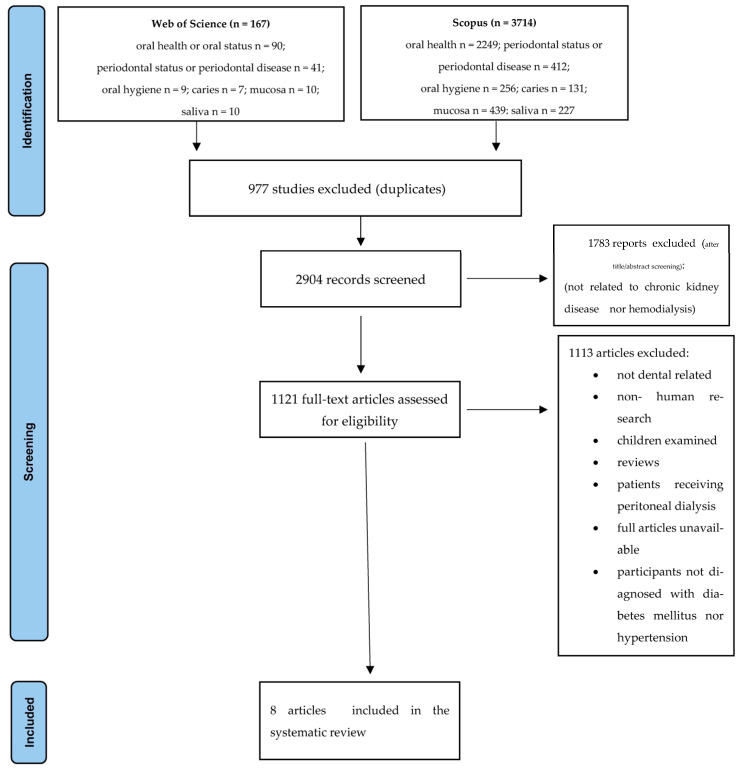
The study selection process presented with the use of the PRISMA flowchart.

The protocol of the research was prepared on the basics of PRISMA guidelines [12,13,14] (Figure 1). The review was not registered. The available literature on the oral cavity status of multimorbid patients receiving hemodialysis was analyzed. SCOPUS and Web of Sciences databases were searched. The inclusion and exclusion criteria were as follows:

Inclusion criteria:

Original articles discussing oral manifestations observed in adult patients (older than 18 y.o.) receiving hemodialysis and diagnosed with diabetes mellitus and/or hypertension, articles written in English, articles published between 1 January 2012 and 17 February 2022, articles with their full text available, articles assessed as satisfactory with the Newcastle–Ottawa Scale.

Exclusion criteria:

Case reports, reviews, or non-human studies.

The MeSH (Medical Subject Headings) indexation was used in order to choose appropriate keywords for database searching. My own experience in the research field resulted in widening the range of keywords and including three that were not available in the MeSH (oral status, periodontal status, caries). As a result, the following terms were used: chronic kidney disease or hemodialysis and oral health or oral status; chronic kidney disease or hemodialysis and periodontal status or periodontal disease; chronic kidney disease or hemodialysis and oral hygiene; chronic kidney disease or hemodialysis and caries; chronic kidney disease or hemodialysis and mucosa; chronic kidney disease or hemodialysis and saliva.

Studies were screened by title and abstract due to the PICO (population, intervention, control, and outcome) criteria [15,16]. The formulated PICO question was as follows: are adult patients who are hemodialyzed and have diabetes mellitus and/or hypertension at an increased risk of developing oral pathologies compared with adult patients who are hemodialyzed or healthy?

The search was conducted on 17 February 2022. Article selections were performed separately by two independent researchers (A.T and D.M.) who were calibrated. The agreement between them was calculated with the usage of Cohen’s Kappa value, which is a commonly used comparative scale [16,17]. The agreement between the reviewers was 0.42 (moderate). The interpretation of Cohen’s Kappa values was as follows:

- <0.00—poor;
- 0.00–0.20—slight;
- 0.21–0.40—fair;
- 0.41–0.60—moderate;
- 0.61–0.80—substantial;
- 0.81–1.00—almost perfect.

### 2.2. Substantive Analysis

The following information was extracted from the chosen studies: the year of the study, the country where it was conducted, the characteristics of both the examined and control groups, the observed status of periodontium, mucosa, saliva, oral hygiene, and the presence of Candida infection. Data were extracted by one researcher (A.T.). The extracted information was checked by another coauthor (M.T.) in order to eliminate the risk of bias.

### 2.3. Quality Assessment

The reliability of these studies was performed with the use of the Newcastle–Ottawa Quality Assessment Scale for cross-sectional studies [18]. This scale enables the quality of manuscripts to be assessed in the following domains: selection (SD), comparability (CD) and outcome (OD). The maximum scoring was 10:5 points in the first category, 2 for the second one, and 3 for the outcome. The interpretation of the Newcastle–Ottawa Quality Scale is as follows:

- 9–10 points: very good quality;
- 7–8 points: good quality;
- 5–6 points: satisfactory quality;
- 0–4 points: unsatisfactory quality [17,18].

This part of the research was conducted independently by two authors (M.R. and D.M.); if any discrepancy occurred, a decision was made by the third author (J.D.).

## 3. Results

### 3.1. Study Characteristics

Searching was conducted in two databases (Web of Science and Scopus); after the duplicates were eliminated, 2904 articles qualified for the first screening (Figure 1). An initial analysis of these articles was performed on the basis of information provided in their titles and abstracts. In the next stage, the chosen publications were analyzed with regard to the inclusion criteria (described in the Section 2). The number of 1783 manuscripts were excluded as they did not discuss either chronic kidney disease or hemodialysis. Next, 1113 articles were rejected as they did not analyze the oral cavity status of patients, no information was provided for at least one additional chronic disease (diabetes mellitus, hypertension), patients under 18 years old were the study participants, research was performed on animals, or patients were on peritoneal dialysis. In addition, if the full text was unavailable, articles or reviews were also rejected. All manuscripts that were rejected fulfilled the exclusion criteria.

Finally, eight articles were included in the review. Two described studies took place in India, one in Iraq, one in Japan, one in Saudi Arabia, and three in Poland. One was published in 2016, one in 2017, two in 2018, two in 2020, and two in 2021 (Table 1). Oral cavity hygiene was described in two articles [1,19], and the next two analyzed the periodontal status of participants [1,20]. The mucosa status and saliva parameters were examined by two groups of researchers [1,21]. Also, two manuscripts described the issue of Candida infections in hemodialyzed patients [19,22].

### 3.2. Oral Hygiene

Three out of eight review articles discussed the problem of oral hygiene in multimorbid hemodialyzed patients and were analyzed. The simplified Oral Hygiene Index by Greene and Vermilion (sOHI) was used as a research tool in each of them; additionally, Trzcionka et al. [25] assessed the Approximal Plaque Index (API) by Lange. Researchers from Poland correlated the information gathered from the intraoral examination with the data obtained from the questionnaire. The results of these studies are presented in Table 2.

### 3.3. Periodontal Status

Researchers from Japan [23] determined the Community Periodontal Index (CPI) to assess periodontitis. They also assessed alveolar bone loss with the use of a Schei ruler.

Swapna et al. [24] checked the periodontal status of their patients by calculating the CPI as well.

Dande et al. [1] in their publication analyzed the periodontal status of participants on the basis of the clinical attachment level (CAL) analysis considering as *periodontitis* the condition where CAL > 1 mm. They also mentioned that they analyzed gingiva recessions, the depth of periodontal pockets, and teeth mobility and furcation involvement; however, they did not present the results of their analysis. They also measured the papilla Bleeding Index by Muhlemann, which they used for the assessment of gingival status (gingivitis).

Trzcionka et al. [20], in order to analyze the periodontal status of the study participants, calculated the following four following indices: Periodontal probing depth (PPD), Clinical Attachment Lost (CAL), Bleeding Index or the Bleeding on probing Index (BI or BOP) and Community Periodontal Index for Treatment Needs (CPITN). On the basics of two indices (PPD and CAL), they divided the patients into three groups with regard to the periodontal status (p1—PPD ≤ 0.5 mm, CAL ≤ 2 mm, healthy periodontium; p2—PPD ≤ 3.5 mm, CAL3–4 mm, specialistic consultation of periodontologist is needed; p3—PPD ≥ 3.5 mm, CAL ≥ 5 mm, specialistic treatment is needed). Table 3 presents the results of the research described included in the review studies.

### 3.4. Mucosa and Saliva

The oral mucosa status of hemodialyzed patients was examined by Swapna et al. [24], Dande et al. [1], and Trzcionka et al. [21]. Researchers from India noted the presence of ulcers, dryness, the uremic fetor, dry-fissured lips, and pale mucosa and gathered information on taste disorders. In the description of the study method, they wrote that they also assessed the unstimulated salivary flow rate with the use of a modified Schirmer’s test; however, they did not present the results of their observations.

Researchers from Poland examined saliva parameters with the usage of the salivary flow rate (stimulated), the buffer capacity of saliva, and its pH. They noted the presence of the following: ulcerations, white and red patches, malformations, candidiasis, ecchymosis, herpes, a geographic tongue, a fissured tongue, the smell of acetone, trauma-related lesions or signs of operations, the overgrowth of gingiva, burning mouth syndrome or pain.

Swapna et al. [24] examined patients in order to note the presence of dry mouth, changes in taste, burning sensations, uremic odor, tongue coatings, mucosal petechiae, ecchymosis, or ulcerations. The results of the cited studies are presented in Table 4.

### 3.5. Oral Candidiasis

The problem of Candida infections was analyzed by three groups of researchers; however the group from Poland analyzed only the presence of mucosal pathologies, while two other groups assessed Candida colonization on the basics of a microbial examination. We decided to include the results of Trzcionka et al. [21] in the part of the review discussing pathologies observed on mucosa.

Ayinampudi et al. [18], in order to conduct their research, gathered samples of whole saliva. With the use of the CHROMagar candida medium, after 72 h, the growth of the following species was examined: Candida albicans, tropicalis, and krusei. The presence of more than five colonies was regarded as positive and confirmed with gram staining for candida—the colonies that did not show positive staining after 72 h were classified as negative.

Al-Sarray et al. [19] gathered the samples with the use of sterile disposable cotton swabs by rubbing the tongue and buccal mucosa. After being transported to the laboratory, the samples were incubated at 35–37 °C for 24–48 h on SDA with chloramphenicol. Candida was identified after gram staining with the usage of a microscope.

The results of Ayinampudi et al. [22] and Al-Sarray et al. [19] are presented in Table 5.

### 3.6. Quality Assessment

The quality assessment of the eight studies included in the review was conducted using the Newcastle–Ottawa Scale. It was performed independently by two researchers (M.R. and D.M.), and the discrepancies were solved by the most experienced member of the research team (J.D.) (Table 6).

To present the results of the assessment, the following numbers were given to particular articles:

1. Ayinampudi et al. [22]
2. Dande et al. [1]
3. Al-Sarray et al. [19]
4. Trzcionka et al. [25]
5. Trzcionka et al. [20]
6. Trzcionka et al. [21]
7. Naruishi et al. [23]
8. Swapna et al. [24]

Articles written by Naruishi et al. [23] and Swapna et al. [24] were assessed as very good, and both obtained nine points out of ten. Three publications written by Polish authors were assessed with eight points, which resulted in very good quality [20,21,25]. Two were assessed as good, each obtaining seven points [1,19]. As far as we are concerned, articles only had a satisfactory quality or above [22]. The assessment of the particular articles by M.R. and D.M. were as follows: articles VII and VIII were classified as very good, articles number IV, V, and VI were classified as good (eight points), article II was assessed as good by A.T and as satisfactory by D.M. Taking into consideration the fact that these differences were considerable, the final assessment was performed by the most experienced member of the research team (J.D.).

## 4. Discussion

The aim of our investigation was to establish evidence of the impact of hypertension and diabetes mellitus on the oral status of hemodialyzed patients who are a major part of our society. It seems important to provide them with appropriate dental care that nowadays seems to be inadequate.

The problem of oral findings in people diagnosed with general diseases is widely discussed in the available literature [23,24,26,27,28,29,30,31]. Observed pathologies are identified in dentition, soft tissues, mucosa, bone, muscles, and even nerves. All the authors of the eight publications included in our review proved the correlation of oral cavity status in the course of general diseases such as end-stage chronic kidney disease, hypertension, and diabetes mellitus [1,19,20,21,22,23,24,25]. Hemodialyzed patients who additionally suffered from the mentioned general diseases were characterized by worse oral status in several aspects—periodontal status, hard tissue condition, and susceptibility to mucosal pathologies. End-stage chronic kidney disease, diabetes mellitus, and hypertension also manifest in the condition of the oral cavity.

The presented review discusses oral findings in hemodialyzed patients diagnosed with diabetes mellitus and/or hypertension published after 2012; however, this issue was assessed earlier. In articles presented before 2018, no information on the influence of hypertension in hemodialyzed patients or on their oral status was found. However, researchers agree that the oral cavity condition of patients diagnosed with end-stage chronic kidney disease and hemodialyzed is worse than in healthy ones [32,33,34,35,36].

The comparability of the examined and control groups between studies included in the review was not satisfactory; in fact, they were impossible to compare directly. The authors decided to prepare the review as an introduction for future research and to improve the methodology of future studies. Our expectations were focused on finding studies that were comparable to our past studies in terms of patient group selection (based on diseases the patient was diagnosed with) and looking for standards in the clinical studies discussing.

Two publications published before 2018 discussed the oral health status of adult hemodialyzed patients diagnosed with diabetes mellitus [24,36]. Teratani et al. compared the oral status of 29 patients with diabetic nephropathy (DN) and 69 people diagnosed with chronic glomerulonephritis (CGN) with a group of 106 non-hemodialyzed patients [36]. They assessed the number of teeth, number of teeth with cavities, periodontal probing depth, clinical attachment loss, bleeding on probing, salivary flow rate, and xerostomy. The authors concluded that DN patients showed worse periodontal and xerostomy parameters in comparison to the CGN and control group. Similar observations were presented by Dande et al., who, despite the fact that there were no statistically significant differences observed, concluded that periodontitis is slightly higher in diabetic hemodialyzed than in non-diabetic individuals [1]. Trzcionka et al. described how, in all groups of hemodialyzed patients, the percentage of people with healthy periodontium was lower, and additionally that the highest percentage of individuals with healthy periodontium among hemodialyzed patients was in those not diagnosed with any other general disease or diagnosed additionally with hypertension [20].

Swapna et al. conducted an oral cavity status assessment in 97 hemodialyzed patients in Bhimavaram Hospital, dividing them into non-diabetic and type 2 diabetic groups [37]. They assessed the pH of unstimulated saliva, CPITN, and DMFT indices. They also noted subjective information (dry mouth, changes in taste, burning sensation) and objective findings (uraemic odor, tongue coating, petechiae, ulcerations, or ecchymosis). They observed an increased pocket depth of 6 mm or more in 23.4% of diabetic patients when compared to non-diabetic individuals (6.00%, *p* = 0.015), which is similar to the findings of the Polish examiners [20]. While assessing the pH, Swapna et al. noted statistically significant differences—a pH value of >7.0 was observed among 34% of non-diabetics and 17% of hemodialized diabetics [24]. Out of all the articles chosen for the review, only Trzcionka et al. assessed the pH of saliva; however, no statistically significant differences were observed [21]. Swapna et al. [24] and Trzcionka et al. [21] also assessed mucosa status. Neither group of researchers observed statistically significant differences in the presence of pain and ulcerations. Dysguesia was statistically more often noted in non-diabetic individuals when compared to diabetic patients in Swapna et al.’s study, while Trzcionka et al. did not observe any statistically significant differences. Similarly, for the uremic fetor, moderately significant differences were observed by Swapna et al. (found in 90% of non-diabetic and 74.5% of diabetics, *p* = 0.044), while Trzcionka et al. did not note any differences [24].

A detailed analysis of the correlations and bilateral dependency between the oral cavity condition and the general condition of the human organism led to the conclusions emphasized by the authors [1,23] that successful treatment, together with the improvement of patients’ performance, may be dependent on oral cavity status. Unfortunately, due to multimorbidity, it might be difficult to define the cause-and-effect dependency. Many proofs have been described to define the relationship between periodontium and the well-being of hemodialyzed patients [1,20,23,24].

The articles included in the review were written by researchers outside Europe, even though more and more people from Europe also suffer due to multimorbidity and are diagnosed with end-stage chronic kidney disease, hypertension, and diabetes mellitus. We realize that the proper analysis of health in that group of patients demands interdisciplinary cooperation [20,21,25].

### Limitations

We had faced a few problems. First of all, a part of our research team was previously engaged in the examination of hemodialyzed patients, which might have caused their bias (A.T, M.T). That is why among the authors, there were other researchers who had never before dealt with the oral status of hemodialyzed patients, including D.M—a researcher with hardly any experience—, M.R—highly experienced in the field of dentistry—and J.D.—an expert in the field of nephrology. The researcher with the least experience (deliberately) was asked to search the databases. A comparison of the results obtained by her with the results obtained by the person who was familiar with the topic resulted in an agreement among the reviewers, which assessed was with the use of Cohen’s Kappa coefficient as moderate (0.42). The differences between the reviewers were then checked by the third author, who was familiar with the topic of the study. The presented results proved that in order to identify the available literature regarding any specific issue, it must be checked if the researchers are familiar with the issue and perfectly understand the inclusion and exclusion criteria.

The risk of bias assessment is a very important aspect of systematic reviews [37,38]. The conduction of that stage in this research in our team led to the conclusion that in order to provide an objective assessment of the risk of bias it was crucial for the person that was not engaged in the research included in the review to prepare that part—which is why we incited professor Duława. What is interesting is the author with the least experience was much stricter in quality assessment than the more experienced one. There is information available that sometimes it might be crucial to contact the authors of the research to perform the risk of bias assessment [38]. We also observed that we needed to prepare an appendix, as sometimes the restrictions of the journal limit the authors in terms of the manuscript volume, so the crucial information must be presented as an appendix.

## 5. Conclusions

There is still a limited number of publications discussing the oral status of hemodialyzed patients diagnosed with hypertension.

It is crucial to analyze a wide range of articles (for instance, in terms of years of publication) in order to prepare a high-quality review. There are hardly any articles combining the systematic review and presentation of results, while in our opinion, this kind of article is the most effective if there are no plans for long-term and multi-stage studies. If multi-stage research is planned, a good systematic review can be a source of information on how to properly prepare the methodology of the study.

There is a necessity to properly organize the research team (the gradation of the experience and engagement in the assessed topic).

The knowledge provided in the included review studies confirms that coexisting diseases (diabetes mellitus) influence the oral cavity status of hemodialyzed patients, causing the deterioration of periodontal status, hygiene, and saliva parameters and making the risk of Candida infections occurrence higher. These facts confirm the necessity for multimorbid patients to be taken care of by an interdisciplinary team of specialists.

## Figures and Tables

**Table 1:** Articles included in the review.

Authors, Title and Year of Publication	Country	Participants/Material	Statistical Analysis
Naruishi et al., “Association between periodontal condition and kidney dysfunction in Japanese adults: A cross-sectional study”; 2016 [23]	Japan	Group DM—48 patients diagnosed with diabetes mellitus Group Dialysis—84 hemodialyzed patients Group Dialysis with DM—32 hemodialyzed people with diabetes mellitus	mean, standard deviation, frequencies, percentages, chi-square test, analysis of variance (ANOVA), Spearman’s rank correlation coefficient, the Turkey- Kramer honest significant difference test Analyses were performed using JMP^®^ 8 ver. 8.0.2 (SAS Institute Japan, Tokyo).
Swapna et al., “Oral health in diabetic and nondiabetic patients with chronic kidney disease”; 2017 [24]	Saudi Arabia	Group A—47 diabetic patients on hemodialysis Group B—54 diabetic patients with chronic kidney disease but not on hemodialysis Group C—50 nondiabetic patients on hemodialysis Group D—43 nondiabetic patients with chronic kidney disease but not hemodialyzed	Chi-square test Analyses were done with the usage of Statistical Package for Social Sciences (SPSS) ver. 15.0 (SPSS Inc., Chicago, IL, USA) and SAS 9.2.
Ayinampudi et al., “Oral Candida colonization in renal disease patients between diabetes and non-diabetes; a comparative study”; 2018 [22]	India	Group I—15 patients diagnosed with chronic kidney disease Group II—15 hemodialyzed patients (for at least 4 months) Group III—renal transplanted patients Each group was divided into diabetic and non-diabetic patients.	percentages, frequencies, risk ratio, and odds ratio
Dande et al., “Oral manifestations in diabetic and nondiabetic chronic renal failure patients receiving hemodialysis”; 2018 [1]	India	144 patients included	percentages, frequencies, means, standard deviation, Pearson chi-square test, Fischer’s exact test, and Student’s test Analyses were performed using the Statistical Package for the Social Sciences ver. 16.0 (SPSS, Chicago, IL, USA).
Al-Sarray et al., “Oral candidiasis in chronic kidney disease”; 2020 [19]	Iraq	50 patients diagnosed with chronic kidney disease	chi-square test and one-way analysis of variance (ANOVA) Analyses were performed with the Statistical Package for the Social Sciences (SPSS) ver. 21.
Trzcionka et al., “Oral cavity status of long-term hemodialyzed patients vs. their socio-economic status”; 2020 [25]	Poland	Examined group: R—hemodialyzed patients (42) R + H—hemodialyzed with hypertension (79) R + D—hemodialyzed with diabetes (16) R + H + D—hemodialyzed with hypertension and diabetes (43) Control group—48 patients not diagnosed with end-stage chronic kidney disease, hypertension, or diabetes	Shapiro–Wilk test, Kruskal–Wallis test, and Mann–Whitney U test Analyses were performed using IBM’s SPSS Statistics 23 program (IBM, Armonk, NY, USA).
Trzcionka et al., “Periodontal treatment needs of hemodialyzed patients”; 2021 [20]	Poland	Examined group: R—hemodialyzed patients (42) R + H—hemodialyzed with hypertension (79) R + D—hemodialyzed with diabetes (16) R + H + D—hemodialyzed with hypertension and diabetes (43) Control group—48 patients not diagnosed with end-stage chronic kidney disease, hypertension, or diabetes	Kruskal–Wallis test, Mann–Whitney U test, test chi-quadrat Analyses were performed using IBM’s SPSS Statistics 23 program (IBM, Armonk, NY, USA).
Trzcionka et al., “Oral mucosa status and saliva parameters of multimorbid adult patients diagnosed with end-stage chronic kidney disease”; 2021 [21]	Poland	Examined group: R—hemodialyzed patients (42) R + H—hemodialyzed with hypertension (79) R + D—hemodialyzed with diabetes (16) R + H + D—hemodialyzed with hypertension and diabetes (43) Control group—48 patients not diagnosed with end-stage chronic kidney disease, hypertension, or diabetes	Kruskal–Wallis test, Mann–Whitney U test and chi-square test Analyses were performed using IBM’s SPSS Statistics 23 program (IBM, Armonk, NY, USA).

**Table 2:** Data regarding oral hygiene presented in the articles included for the review.

Reference	Group	Result	Conclusions
Naruishi et al. [23]	Hemodialyzed with diabetes mellitus	Not presented	No significant differences in the OHI score among the groups (*p* = 0.84, hemodialysis vs. hemodialysis with DM).
Dande et al. [1]	Hemodialyzed non-diabetic patients	Poor OHI 54.05% 40/74	*p* = 0.000 The diabetic group revealed significantly higher levels of poor oral hygiene
	Hemodialyzed with diabetes mellitus	poor OHI 88.57% 62/70	
Trzcionka et al. [25]	Hemodialyzed	API = 74.55 OHI-S = 1.70	The statistical analysis of s OHI with the usage of the Kruskall–Wallis test showed statistically significant differences (*p* < 0.001). Test Mann–Whitney U proved that s OHI values were significantly lower in healthy patients and higher in hemodialyzed patients with diabetes mellitus and hypertension than in hemodialyzed and hemodialyzed with hypertension and higher in hemodialyzed with diabetes mellitus than in hemodialyzed. Kruskall–Wallis test also showed statistically significant differences in API values (*p* < 0.001)—the control group presented significantly lower values and additionally hemodialyzed with diabetes and hypertension than only hemodialyzed.
	Hemodialyzed with hypertension	API = 69.40 OHI-S = 2.27	
	Hemodialyzed with diabetes mellitus	API = 95.71 OHI-S = 3.11	
	Hemodialyzed with hypertension and diabetes mellitus	API = 85.63 OHI-S = 3.64	
	Control group	API = 26.68 OHI-S = 1.11	

**Table 3:** Data regarding periodontal status presented in the articles included to review.

Reference	Group	Results	Conclusions
Naruishi et al. [23]	Hemodialysis vs. hemodialysis + diabetes mellitus	Not presented	No significant differences among the groups with regard to alveolar bone loss (it tended to be higher in dialysis with DM in comparison to dialysis; *p* = 0.079) CPI—lower in the DM group than in others (*p* = 0.083 vs. hemodialysis with DM; *p* = 0.033 vs. hemodialysis)
Swapna et al. [24]	Hemodialysis + diabetes mellitus	CPI = 3.1	In diabetics with chronic kidney disease, an increased periodontal pocket depth was observed in comparison to nondiabetics (*p* < 0.05).
	Diabetics + chronic kidney disease not on hemodialysis	CPI = 3.0	
	Nondiabetics on hemodialysis	CPI = 2.8	
	Nondiabetics not on hemodialysis	CPI = 2.8	
Dande et al. [1]	Hemodialyzed non-diabetic patients	Gingivitis: 25.71% (18/74) periodontitis: 13.51% (10/74)	Gingivitis (*p* = 0.531) and periodontitis (*p* = 0.191) showed no statistically significant differences, but the tendency to be slightly higher in diabetics was observed.
	Hemodialyzed with diabetes mellitus	Gingivitis: 32.43% (24/70) periodontitis: 25.71% (18/70)	
Trzcionka et al. [20]	Hemodialyzed	BI—M = 49.61 CPI0—6%, CPI1—21%, CPI2—39%, CPI3—9%, CPI4—24% TNI—28%, TNII—48%, TNIII—24%	The Kruskall–Wallis test showed that the BI value in the control group was significantly lower (*p* < 0.001). In the control group, there were significantly more patients qualified for CPI1 and CPI2 and less for CPI3 and CPI4 than in the hemodialyzed people. Most of the patients from the control group were qualified for TNIII, and most were from the hemodialyzed TNII. P1 (*p* = 0.000) P2 (*p* = 0.533) P3 (*p* = 0.000) In all subgroups of the hemodialyzed patients, the percentage of people with healthy periodontium was significantly lower. In the examined patients, the highest percentage of patients with healthy periodontium was in hemodialyzed patients and hemodialyzed patients with hypertension.
	Hemodialyzed with hypertension	BI—M = 44.73 CPI0—6%, CPI1—30%, CPI2—30%, CPI3—13%, CPI4—22% TNI-35%, TNII—42%, TNIII—22%	
	Hemodialyzed with diabetes mellitus	BI—M = 54.00 CPI0—0%, CPI1—11%, CPI2—67%, CPI3—0%, CPI4—22% TNI—11%, TNII—67%, TNIII—22%	
	Hemodialyzed with hypertension and diabetes mellitus	BI—M = 37.55 CPI0—0%, CPI1—10%, CPI2—41%, CPI3—21%, CPI4—28% TNI—10%, TNII—62%, TNIII—28%	
	Control group	BI—M = 5.36 CPI0—28%, CPI1—30%, CPI2—21%, CPI3—13%, CPI4—9% TNI—57%, TNII—34%, TNIII—9%	

**Table 4:** Data regarding mucosa status and saliva parameters presented in the articles included to the review.

Reference	Group	Result	Conclusion
Swapna et al. [24]	Hemodialysis + diabetes mellitus	subjective dry mouth: 37/47 subjective dysgeusia: 37/47 mucosal pain:14/47 uremic odor: 35/47 tongue coating: 18/47 mucosal petechiae: 15/47 ecchymosis:0 mouth ulceration: 1/47 dry mouth: 47/47	Dysgeusia was significantly more prevalent in hemodialyzed nondiabetics (*p* = 0.03). Statistically significant differences were also observed in the frequency of occurrence of uremic odor (*p* = 0.04) and mucosal petechiae (*p* = 0.01).
	Diabetics + chronic kidney disease not on hemodialysis	subjective dry mouth: 40/54 subjective dysgeusia: 40/54 mucosal pain:17/54 uremic odor: 41/54 tongue coating: 18/54 mucosal petechiae: 15/54 ecchymosis:0 mouth ulceration: 1/54 dry mouth: 53/54	
	Nondiabetics on hemodialysis	subjective dry mouth: 31/50 subjective dysgeusia: 45/50 mucosal pain:18/50 uremic odor: 45/50 tongue coating: 9/50 mucosal petechiae: 5/50 ecchymosis:0 mouth ulceration: 0 dry mouth: 48/50	
	Nondiabetics not on hemodialysis	subjective dry mouth: 28/43 subjective dysgeusia: 28/43 mucosal pain:15/43 uremic odor: 39/43 tongue coating: 9/43 mucosal petechiae: 5/43 ecchymosis:0 mouth ulceration: 0 dry mouth: 42/43	
Dande et al. [1]	Hemodialyzed non-diabetic patients	ulcers: 8.10%, dryness: 48.64%, uremic fetor: 59.45%, dry-fissured lips: 2.70%, pale mucosa: 35.13%, unpleasant taste: 35.13%	In diabetic patients, significantly more patients were diagnosed with a uremic fetor (*p* = 0.005), unpleasant taste (*p* = 0.009), dry-fissured lips (*p* = 0.002), and pale mucosa (*p* = 0.019).
	Hemodialyzed with diabetes mellitus	ulcers: 8.77%, dryness: 60.00%, uremic fetor: 88.57%, dry-fissured lips: 28.57%, pale mucosa: 62.85%, unpleasant taste: 65.71%	
Trzcionka et al. [21]	Hemodialyzed	S1 = 0.55 mL/min S2 = 0.72 mL/min Buffer capacity: VL—21.4%, L—14.3%, N—64.3% pH = 6.39 dryness: 50%, ecchymosis: 36%, candidiasis: 40%, fissured tongue: 33%, trauma-related oral lesions: 21%, ulcerations: 0, herpes simplex: 0, overgrowth of gingiva: 0, signs of operations: 0, malformations of mucosa: 36%, white patches: 17%, taste disorders: 10%, geographic tongue: 7%, halitosis: 7%, red patches: 5%, pain: 2%, burning mouth syndrome: 2%	The salivary flow rate after hemodialysis was significantly higher in healthy participants (*p* < 0.001). The chi-squared test showed statistically significant differences (*p* < 0.05) in healthy people and showed fewer participants with a very low buffer capacity. There were no statistically significant differences in pH values (*p* = 0.987). The percentage of healthy patients who complained about dryness was significantly lower (*p* = 0.002); in that group of patients, the percentage of patients with ecchymosis (*p* = 0.005), candidiasis (*p* = 0.003), fissured tongue (*p* = 0.000) and trauma-related oral lesions (*p* = 0.021) was also lower.
	Hemodialyzed with hypertension	S1 = 0.63 mL/min S2 = 0.68 mL/min Buffer capacity: VL—31.6%, L—16.5%, N—51.9% pH = 6.22 dryness: 54%, ecchymosis: 19%, candidiasis: 37%, fissured tongue: 40%, trauma-related oral lesions: 16%, ulcerations: 1%, herpes simplex: 1%, overgrowth of gingiva: 1%, signs of operations: 1%, malformations of mucosa: 19%, white patches: 19%, taste disorders: 10%, geographic tongue: 5%, halitosis: 5%, red patches: 8%, pain: 4%, burning mouth syndrome: 1%	
	Hemodialyzed with diabetes mellitus	S1 = 0.40 mL/min S2 = 0.80 mL/min Buffer capacity: VL—12.5%, L—18.8%, N—68.8% pH = 5.96 dryness: 50%, ecchymosis: 31%, candidiasis: 31%, fissured tongue: 25%, trauma-related oral lesions: 25%, ulcerations: 0, herpes simplex: 0, overgrowth of gingiva: 6%, signs of operations: 0, malformations of mucosa: 36%, white patches: 12%, taste disorders: 19%, geographic tongue: 6%, halitosis: 12%, red patches: 5%, pain: 0, burning mouth syndrome: 0	
	Hemodialyzed with hypertension and diabetes mellitus	S1 = 0.55 mL/min S2 = 0.86 mL/min Buffer capacity: VL—23.3%, L—14%, N—62.8% pH = 6.3 dryness: 48%, ecchymosis: 39%, candidiasis: 39%, fissured tongue: 39%, trauma-related oral lesions: 16%, ulcerations: 0, herpes simplex: 0, overgrowth of gingiva: 0, signs of operations: 0, malformations of mucosa: 40%, white patches: 23%, taste disorders: 10%, geographic tongue: 7%, halitosis: 2%, red patches: 12%, pain: 2%, burning mouth syndrome: 5%	
	Control group	S = 1.55 mL/min Buffer capacity: VL—2.1%, L—25%, N—72.9% pH = 7.00 dryness: 19%, ecchymosis: 10%, candidiasis: 8%, fissured tongue: 2%, trauma-related oral lesions: 0, ulcerations: 4%, herpes simplex: 6%, overgrowth of gingiva: 0, signs of operations: 0, malformations of mucosa: 10%, white patches: 8%, taste disorders: 6%, geographic tongue: 2%, halitosis: 0, red patches: 8%, pain: 0, burning mouth syndrome: 0	

S1—stimulated saliva secretion before hemodialysis, S2—stimulated saliva secretion after hemodialysis, VL—very low, L—low, N—normal.

**Table 5:** Data regarding Candida presented in the articles included to review.

Reference	Group (n)	Results	Conclusions
Ayinampudi et al. [22]	Hemodialyzed non-diabetic patients (11)	Candida observed in 3 samples (27%)	The high-risk ratio (1.774) is an indication that the presence of Candida is probably higher in diabetic patients.
	Hemodialyzed diabetic patients (4)	Candida observed in 1 sample (25%)	
Al-Sarray et al. [19]	Hemodialyzed (4)	Candida observed in 2 samples (50%)	Diabetes mellitus and hypertension do not predispose patients to oral Candidiasis.
	Hemodialyzed with hypertension (33)	Candida observed in 15 samples (45.5%)	
	Hemodialyzed with diabetes mellitus (1)	Candida positive	
	Hemodialyzed with hypertension and diabetes mellitus (12)	Candida observed in 6 samples (50%)	

n—number of patients in a particular group.

**Table 6:** Quality assessment with the Newcastle–Ottawa Scale.

Study Number	Selection				Comparability	Outcome		Quality
	A	B	C	D		A	B	
1.	1	0	0	0	2	2	0	SATISFACTORY
2.	1	0	1	0	2	2	1	GOOD
3.	1	1	0	0	2	2	1	GOOD
4.	1	1	1	1	1	2	1	GOOD
5.	1	1	1	1	1	2	1	GOOD
6.	1	1	1	1	1	2	1	GOOD
7.	1	1	1	1	2	2	1	VERY GOOD
8.	1	1	1	1	2	2	1	VERY GOOD

## Data Availability

Not applicable.
